# Risk Factor Analysis for Infection after Medial Open Wedge High Tibial Osteotomy

**DOI:** 10.3390/jcm10081727

**Published:** 2021-04-16

**Authors:** Ta-Wei Liu, Chih-Hao Chiu, Alvin Chao-Yu Chen, Shih-Sheng Chang, Yi-Sheng Chan

**Affiliations:** 1 Department of Orthopedic Surgery, Chang Gung Memorial Hospital, Linkou 333, Taiwan; davidliu2057@cgmh.org.tw (T.-W.L.); alvinchen@cgmh.org.tw (A.C.-Y.C.); 2 Bone and Joint Research Center, Chang Gung Memorial Hospital, Linkou 333, Taiwan; joechiu0115@gmail.com; 3 Department of Orthopedic Surgery, Chang Gung Memorial Hospital, Taoyuan 333, Taiwan

**Keywords:** high tibial osteotomy, infection, risk factor, arthroscope

## Abstract

Background: Medial open wedge high tibial osteotomy (MOWHTO) is a well-established treatment for osteoarthritis of the medial tibiofemoral compartment. Surgical site infection (SSI) after MOWHTO is a devastating complication that may require further surgery. In this study, we aimed to identify the risk factors for infection after MOWHTO over 1 to 4 years of follow-up. Methods: Fifty-nine patients who underwent MOWHTO combined with knee arthroscopic surgery were included in this prospective study. Artificial bone grafts were used in all cases. Possible risk factors, including sex, age, body mass index (BMI), underlying disease, hospitalization length, correction angle, and surgery time, were recorded. Both univariate and multivariate analysis were used. Results: A total of 59 patients who underwent 61 operations were included. Eleven patients (18.0%) were reported to have SSI. Univariate analysis showed that smoking and diabetes mellitus were positively associated with SSI. Multivariate analysis showed that smoking and age were positively associated with SSI. Three patients (4.9%) were reported to suffer from deep SSI, requiring surgical debridement, all of whom were male smokers. Conclusion: Smoking, diabetes mellitus, and old age were identified to be possible risk factors of SSI after MOWHTO. These findings are common risk factors of SSI after orthopedic surgery according to the literature. Patient selection should be performed cautiously, and postoperative prognosis for MOWHTO should be carefully explained to patients who smoke.

## 1. Background

Medial open wedge high tibial osteotomy (MOWHTO) is a well-established surgical method for knee osteoarthritis of the medial compartment with genu varum deformity. The procedure alleviates the weight-bearing load from the medial compartment and corrects malalignment [1,2,3]. MOWHTO has been shown to have high survivorship and is able to improve quality of life, pain, and knee range of motion according to long-term follow-up [4]. MOWHTO combined with arthroscopic knee surgery for cartilage restoration, ligament reconstruction, and meniscus repair facilitates the healing process and has become a recent trend [5,6].

Complications after MOWHTO have been reported in the literature, including superficial and deep SSI, delayed union and nonunion, hardware failure, hematoma, neuropathy, and lateral cortex fracture. [7] Postoperative deep SSI has been regarded as one of the most detrimental complications, which may lead to multiple revision surgeries, loss of correction degree, and nonunion. [8] However, few past reports have focused on this issue. The existing papers on the topic differ based on study design, patient collectives, implant selection, surgical technique, and investigation parameters. In this study, we present a case series of patients with the same surgical technique and implant. We aim to identify the risk factors for infection after MOWHTO combined with arthroscopic procedures. Identifying these risk factors will help surgeons prevent SSI in the future.

## 2. Methods

From June 2016 to May 2019, we prospectively enrolled 59 consecutive patients (20 males and 39 females) who underwent MOWHTO with a knee arthroscopic procedure. All patients signed informed consent forms for institutional review board approval before their surgery and underwent the same treatment protocols. All preoperative evaluations and surgical procedures were performed by a single surgeon.

### 2.1. Patient Selection

Patient selection was based on the guidelines developed by the ISAKOS (International Society of Arthroscopy, Knee Surgery and Orthopaedic Sports Medicine) in 2004. Inclusion criteria for MOWHTO included age between 40 to 70, isolated medial compartment arthrosis, metaphyseal varus deformity, normal range of motion, and normal ligament stability. Exclusion criteria included bicompartmental arthrosis, patellofemoral arthrosis, BMI > 30, and flexion contracture >15 degrees.

### 2.2. Surgical Technique

Preoperative surgical planning was undertaken according to Miniaci’s method [9]. The operation was performed under general anesthesia. A tourniquet was applied in all cases. The leg was disinfected with povidone-iodine soap and povidone-iodine alcoholic solution. The leg was draped free with non-disposable fabric draping material. Arthroscopic examination was performed first in all patients using the standard anteromedial and anterolateral portals. Chondral resurfacing using the microfracture technique was performed in all patients. Meniscal repair using Fastfix or partial meniscectomy was performed as needed. A reverse L-shaped incision was used for MOWHTO. The osteotomy was completed according to the surgical technique originally mentioned by Staubli et al. [10]. The direction of the osteotomy in the coronal plane was marked with 2.4 mm K-wire and checked under fluoroscopic examination. The osteotomy was performed in a biplanar fashion. The first osteotomy was performed distal to the K-wire, parallel to the tibial slope. The second osteotomy started in the anterior third of the proximal tibia at an angle of 135° to the first osteotomy plane and exited the bone proximal to the insertion of the patellar tendon. The osteotomy was opened by stepwise insertion of osteotomes. The mechanical axis was then adjusted according to the preoperative planning, and the position was maintained with a spreader. A standard Tomofix (Synthes) locking plate was used as the fixation device. To facilitate bone healing, an artificial bone graft with Stimulan (Biocomposites, Keele, UK) was filled in the medial osteotomy site in all patients. A drainage tube with Hemovac was placed in all patients and was typically removed on postoperative day 2. Prophylactic antibiotics with 1 gram of cefazolin within 30 min preoperatively and cefazolin 1g Q8H for one day were given in all cases.

### 2.3. Rehabilitation Protocol

Partial weight bearing was allowed immediately after the surgery. No limitation in the range of motion was needed; therefore, the patients were not recommended to wear braces. Progression to full weight bearing was allowed six weeks after surgery, as tolerated by the patient.

### 2.4. Definition of Superficial and Deep SSI

Superficial SSI was defined as infection within 30 days of the surgery that involved only the skin and subcutaneous tissue. Local tenderness, swelling, erythematous change, and heat detected at outpatient department follow up suggested the occurrence of superficial SSI, and empirical antibiotics with cefadroxil were given for one week. If the patient also presented with chillness, fever, or pus discharge, deep SSI was suspected, and a blood test was done. Elevated CRP and WBC levels would further support the diagnosis, and debridement surgery would be arranged. Deep surgical site infection is defined in this series as cases that underwent surgical debridement.

### 2.5. Statistical Ananlysis

Data regarding demographic characteristics, including age, body mass index (BMI), patient comorbidities, and smoking, as well as surgery-related factors, including hospitalization length, correction angle, meniscus repair, and surgery time, were collected. Univariate analysis was carried out using cross-tabulations with the Fisher’s exact test for categorical variables and independent *t*-test for numerical variables. Multivariate analysis was carried out using a backward stepwise logistic regression model. Odds ratios (ORs) and 95% confidence intervals (CIs) were used to identify significant predictors of SSI. Significance was defined as a *p*-value < 0.05. All statistical analyses were performed using the SPSS software package (ver. 25).

## 3. Results

A total of 59 patients who underwent 61 procedures were included in this study. The overall demographic data and surgery-related data are presented in Table 1.

**Figure 1:**
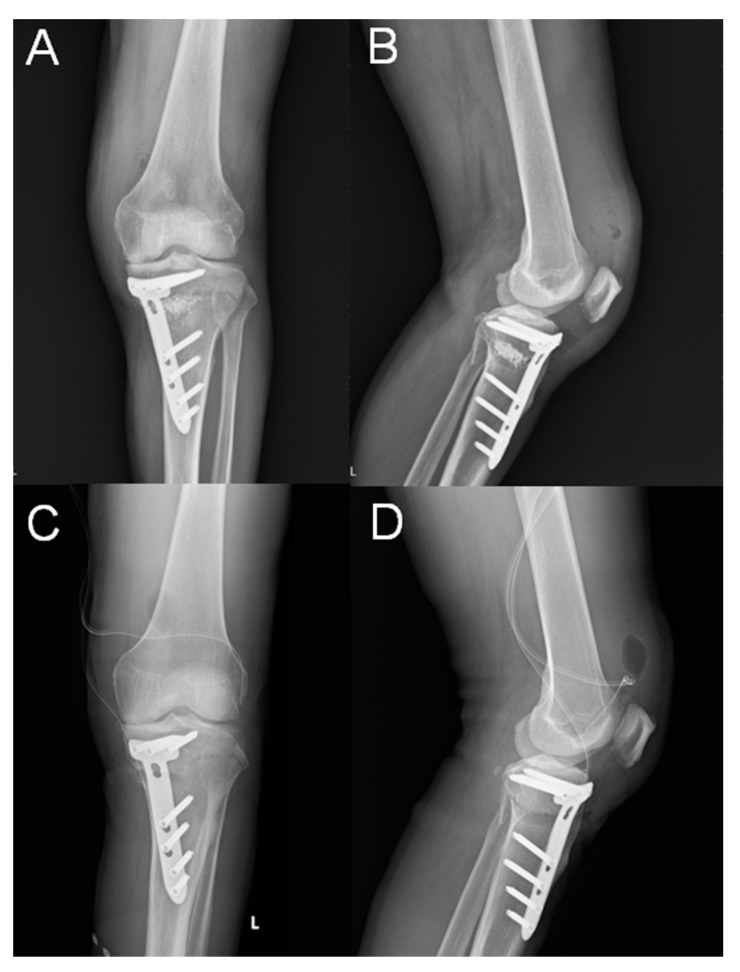
(**A**,**B**) A 58-year-old male presented with an acute deep SSI 2 weeks after MOWHTO. Plain radiographs with anteroposterior and lateral views showed that the Tomofix locking plate position was slightly more anterior than standard position, and the artificial bone graft could be seen at the osteotomy site. (**C**,**D**) After debridement surgery, the artificial bone graft was removed, and a drainage tube was placed in the knee joint and at the osteotomy site.

**Figure 2:**
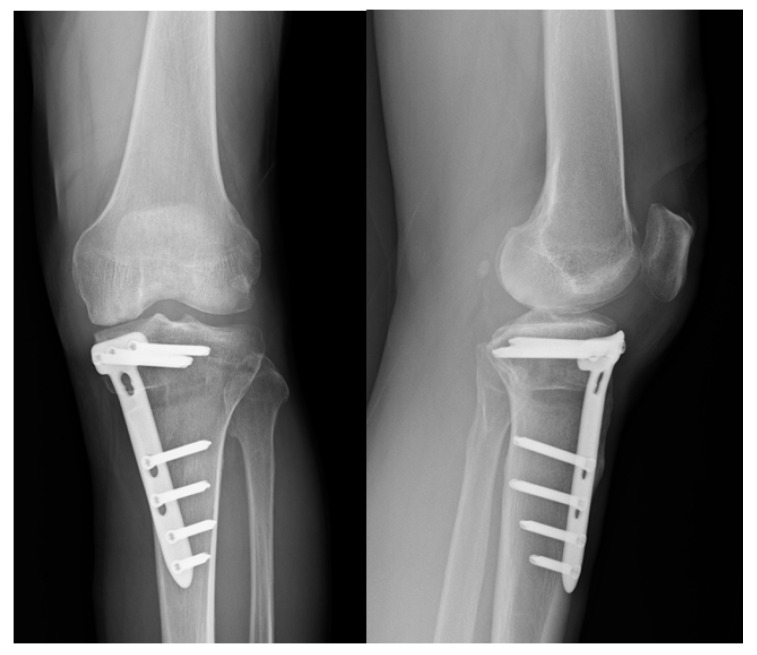
Plain radiographs with anteroposterior and lateral views at the 8-month follow-up showed solid bony union.

Eight cases of superficial SSI developed; all of those were treated successfully with oral antibiotics for one week. Three cases of deep SSI occurred in our series (Figure 1). One occurred 2 weeks after the operation, and the other two were delayed, taking place 2 months and 3 months, respectively, after the operation. These cases were treated with arthroscopic debridement of knee joint and open debridement of the previous osteotomy site, with removal of the artificial bone graft. A drainage tube with hemovac was placed both in the knee joint and at the osteotomy site. The implants were retained. Cases of deep surgical site infection are presented in Table 2. Parenteral antibiotics were given for 6 weeks. All cases were treated successfully with preservation of plate fixation, and all reached bony union after 8 months of follow-up (Figure 2). The incidence of overall SSI in our series was 18.0%, in which 13.1% were superficial, and 4.9% were deep SSI.

### 3.1. Univariate Analysis

Patients in the SSI group had a significantly higher incidence of being a smoker (36.4% vs. 4%, *p* = 0.008) and having type 2 diabetes mellitus (27.3% vs. 4%, *p* = 0.037). There was a considerable trend toward significance in the age between two groups (62.1 y/o vs. 58 y/o, *p* = 0.073). BMI, preoperative IKDC (International Knee Documentation Committee) score, and hypertension failed to show significant differences between groups. There was no significant difference between groups in hospitalization length, correction angle, surgery time, or meniscus repair percentage (Table 3).

### 3.2. Multivariate Analysis

**Figure 3:**
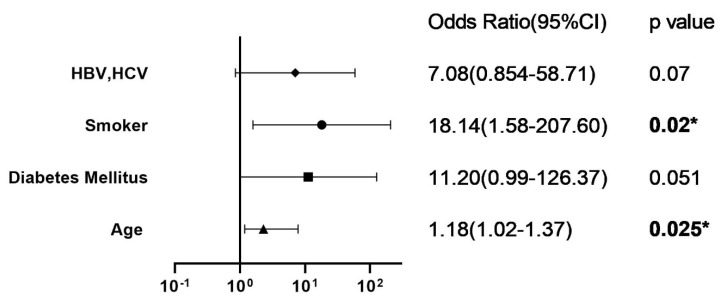
Multivariate analysis. HBV: Hepatitis B; *: statistically significant.

Four variables, including smoking, hepatitis B and C, age, and diabetes mellitus, were selected using the backward logistic regression method. In this model, SSI after MOWHTO was positively associated with smoking (OR 18.14, 95% CI 1.58–207.6) and age (OR 1.18, 95% CI 1.02–1.37) (Figure 3).

## 4. Discussion

The incidence of overall SSI in our series was 18.0%; of that, 13.1% were superficial and 4.9% were deep SSI. In a systematic review that included 26 studies (level II: 1, level III: 5, and level IV: 20) and a total of 2026 patients, performed by Anagnostakos et al. [11] in 2013, the incidence of superficial SSI was estimated to be 1–9% and that of deep SSI to be 0.5–4.7%. However, in the national database study by Kawata et al., the incidence of SSI was 1.52% and that of deep SSI was 0.39% [12]. Our number was closer to that described by Anagnostakos, but ours was much higher than the number in the latter article.

In our study, univariate analysis showed smoking and diabetes mellitus were positively associated with SSI. Multivariate analysis showed that smoking and age were positively associated with SSI. In recent research by Kawata et al. [13] using the national database in Japan, smoking, male sex, and longer anesthesia time were found to be associated with a higher incidence of SSI. Younger age was a protective factor. This is the only study with a large patient cohort (12,853 patients, 195 cases of infection) targeting this issue, and their findings were close to ours.

In our study, all the patients who developed deep SSI were male. Male sex has been reported to be associated with periprosthetic joint infection after total knee arthroplasty and total hip arthroplasty in the literature [14,15]. In a recent database analysis in Germany, male sex was found to be a significant risk factor for surgical site infection after orthopedic procedures [14]. The biological differences between aged males and females have been studied. These studies have led to a focus on estrogen. Estrogen affects wound healing by regulating genes associated with regeneration, matrix production, protease inhibition, and epidermal function. Estrogen has been found to improve wound healing in aged individuals, whereas androgen has a negative impact [16].

Seo et al. [17] and Houten et al. [18] have reported smoking to be associated with delayed union and nonunion after MOWHTO. The negative impact of smoking on fracture healing and the increased risk of infection after trauma or other orthopedic surgeries have been well established in the past [19,20,21,22]. Our data refute the finding that being a nonsmoker is associated with infection after HTO, which was outlined in an article by Dahl et al. [23] on the hemicallotasis technique. We believe that same is true of other orthopedic surgeries, and it is important that patients be advised to stop smoking before undergoing MOWHTO.

Old age is associated with delayed wound healing, which could potentially lead to SSIs. Past studies in the literature have reported old age as a risk factor for infections after orthopedic surgery [24,25,26]. In an interesting study, Kaye et al. [27] found the risk of SSI to increase by 1.1% per year between the ages of 17 and 65 years; however, at age ≥65 years, risk decreased by 1.2% per year. Of note, the majority of cases undergoing MOWHTO are under the age of 65.

In the review by Anagnostakos et al. [28], the possible surgery-related risk factors for infection after high tibial osteotomy identified in the literature included oblique (L-shaped) skin incision, insertion of artificial bone grafts, and one day of hospitalization. Notably, most studies had a level of evidence of IV and a small number of patients, and only one level II study could be found. We have not compared different surgical methods, and other surgery-related factors, including surgery time, meniscus repair, and correction angle, had negative findings.

Our experience is that artificial bone grafts can facilitate bone healing, and there has been report of safe usage of artificial bone grafts in the past [28,29,30]. However, concern regarding the insertion of artificial bone grafts at the osteotomy site was brought forward by Spahn [31] in his series of 85 patients. Deep SSI only developed in patients in whom artificial bone grafts were used. This finding was supported by the national database analysis by Kawata et al. [12]. Multivariable analysis in their study showed SSI to be positively associated with the use of artificial bone grafts versus natural bone grafts. This may explain why our rate of SSI was higher than that in the past literature. The influence of artificial bone is a possibility, and further studies on the use of artificial bone grafts are needed.

The management of deep SSI after MOWHTO reported in the past literature included the removal of internal fixation devices, debridement, and intravenous antibiotic injection. Some authors have proposed antibiotic-impregnated calcium phosphate cement insertion [8,32,33]. However, hardware removal can be problematic before bony union. In our series, we successfully treated three cases of deep SSI with thorough debridement, hardware retention, and intravenous antibiotics. However, the success rate of this procedure requires further investigation.

Our study identified three possible demographic risk factors, smoking, diabetes mellitus, and old age, to be associated with SSI after MOWHTO. These findings correlate well with the past literature. The main limitation to our research was the relatively small patient number, which may lead to a higher possibility of bias. Larger studies are needed to better support our results. We also lacked the use of different types of osteotomy, skin incisions, and implant types in order to compare between groups. However, this also brings about the advantage of consistency in surgical technique. Our rate of SSI was higher than in past literature; this may be explained by the use of artificial bone grafts.

## 5. Conclusions

Smoking, diabetes mellitus, and old age were identified to be possible risk factors of SSI after MOWHTO. These findings are common risk factors of SSIs after orthopedic surgery in the literature. Patient selection should be performed cautiously, and postoperative prognosis for MOWHTO should be carefully explained to patients with multiple risk factors. The use of artificial bone grafts should also be carefully considered.

## Figures and Tables

**Table 1:** Demographic data.

Parameter	Total
Number of procedures	61
Number of patients	59
Side, R:L	35:26
Sex, M:F	20:39
Age (years), mean (SD)	58.7 ± 6.9
Body mass index (kg/m^2^), mean (SD)	27.2 ± 3.6
Correction (degree), mean (SD)	10 ± 3
Meniscus repair (number)	43
Operation time (minutes), mean (SD)	125 ± 27
Hospitalization length (days), mean (SD)	5.2 ± 0.8

R: right; L: left; M: male; F: female.

**Table 2:** Cases of deep surgical site infection.

Sex, Age, BMI	Comorbi-dities	Smoking	Correction Angle	Intraoperative Complications	Time to Infection	Culture	Treatment	Time to Union
M, 64, 25.3	HCV	yes	17 degree	nil	3 months	No growth	Arthroscopic debride of knee joint, open debride of osteotomy site tics with daptomycin and Ceftazidime for a total of 6 weeks	8 months
M, 58, 26.1	Type II DM	yes	9 degree	nil	2 weeks	Staphylococcus epidermidis	Arthroscopic debride of knee joint, open debride of osteotomy site, systemic antibiotics with daptomycin for total of 6 weeks	8 months
M, 46, 26.4	Nil	yes	11 degree	Lateral hinge fracture, Takeuchi type III, fixation with cannulated screws × 2	2 months	MSSA	Open debride of osteotomy site, systemic antibiotics with Oxaxillin for a total of 6 weeks	8 months

M: male; HCV: Hepatitis C, Nil: No comorbidities, DM: Diabetes Mellitus, MSSA: methicillin-susceptible Staphylococcus aureus.

**Table 3:** Univariate analysis.

Parameter	No Infection *n* = 50	Surgical Site Infection *n* = 11	*p*-Value
Sex, male	15 (30%)	5 (45.5%)	0.479
Age, mean	58	62.1	0.073
BMI, mean	27.4	26.3	0.337
Preop IKDC	44.2	45.5	0.834
Smoker	2 (4%)	4 (36.4%)	0.008 *
Diabetes mellitus	2 (4%)	3 (27.3%)	0.037 *
Hypertension, *n*	12 (24%)	4 (36.4%)	0.457
Hepatitis B, C, *n*	5 (10%)	3 (27.3%)	0.148
Days of hospitalization	5.2	5.5	0.336
Correction angle	10	9.8	0.811
Surgery time, min	127.1	117	0.274
Meniscus repair	34 (68%)	10 (91%)	0.415

Bold with *: statistically significant; BMI: body mass index; IKDC: International Knee Documentation Committee.

## Data Availability

Data is in private clinical notes recorded in Chang Gung Memorial Hospital.

## Abbreviations

MOWHTO	Medial open wedge high tibial osteotomy
SSI	Surgical site infection
